# Chromosome segregation in a minimal bacterial cell driven by SMC protein complexes

**DOI:** 10.1002/pro.70604

**Published:** 2026-05-05

**Authors:** Andrew K. Maytin, Benjamin R. Gilbert, Zaida Luthey‐Schulten

**Affiliations:** ^1^ Department of Physics University of Illinois at Urbana Champaign Urbana Illinois USA; ^2^ Science and Technology Center for Quantitative Cell Biology University of Illinois at Urbana Champaign Urbana Illinois USA; ^3^ Department of Chemistry University of Illinois at Urbana Champaign Urbana Illinois USA

**Keywords:** chromosome replication, chromosome segregation, SMC proteins, topoisomerase

## Abstract

Minimal bacterial cells such as JCVI‐Syn3A provide a powerful system for uncovering the essential mechanisms of chromosome organization and segregation. Lacking canonical systems such as Min and ParABS, JCVI‐Syn3A relies primarily on structural maintenance of chromosomes (SMC) protein complexes for partitioning. Here, we investigate a four‐dimensional (4D; three spatial dimensions plus time) polymer‐based model of the JCVI‐Syn3A chromosome (543 kbp) that captures replication and partitioning dynamics across the full cell cycle. Our simulations reproduce chromosome segregation mediated by SMC‐driven loop extrusion and reveal how segregation depends on the number of SMC complexes, their translocation speed, and their dwell time on DNA. A systematic parameter scan shows that segregation is strongly predicted by the effective loop coverage, which represents the expected fraction of the chromosome extruded into loops. We generate contact maps for stationary‐phase cells to directly connect our simulations with 3C experiments, and for replicating chromosomes throughout the cell cycle to provide new, testable predictions for synchronized cell populations. Our results suggest that SMC protein complexes and topoisomerases can drive chromosome segregation in minimal cells without additional partitioning systems provided loop extrusion achieves sufficient genomic coverage.

## 1 INTRODUCTION

Chromosome organization is more readily understood when it can be visualized in three dimensions rather than inferred indirectly from experiments. Computational modeling now makes such visualization possible, providing dynamic, spatially resolved views of processes that cannot be easily observed in living cells (Rose et al., 2024, Goodsell & Autin, 2024, Thornburg et al., 2026). This capability is especially valuable in bacteria, where a small number of Smc–ScpAB complexes must coordinate large‐scale chromosome motions (Kim et al., 2023).

Recent advances in our understanding of bacterial chromosome segregation have brought us to a point where constructing well‐informed models is feasible. While the relative contributions of entropy, DNA loop extrusion, origin‐specific systems such as ParABS, and ribosome distributions are still being clarified, progress is being made in identifying the conditions under which different combinations of these mechanisms are sufficient to drive segregation (Gogou et al., 2021; Harju et al., 2024; Papagiannakis et al., 2025). Recent studies have combined Hi‐C contact maps with an energy landscape framework, revealing how structural maintenance of chromosomes (SMC) activity expands the physical regime permitting faithful segregation (Brahmachari et al., 2024).

In tandem, the structural and mechanistic picture of SMC complexes has sharpened. Experiments from the Dekker group have quantified key loop‐extrusion parameters such as step size (Ryu et al., 2021), extrusion speed (Ganji et al., 2018; Nomidis et al., 2022), direction switching (Barth et al., 2025), and supercoil interactions (Kim et al., 2022). While most work has focused on eukaryotic condensin, recent bacterial studies have directly visualized loop extrusion and investigated the stoichiometry of Smc–ScpAB (Rumyantseva et al., 2025). Efforts to elucidate the molecular mechanism underlying loop extrusion are ongoing (Moon & Ryu, 2023), with recent studies providing important insights into the physical constraints and energetics of loop extrusion (Bonato et al., 2025). Simulations from the Mirny group have shed light on chromosome partitioning dynamics and enabled inference of SMC binding/unbinding and blocking/bypassing parameters from *Bacillus subtilis* Hi‐C data (Banigan et al., 2020; Brandão et al., 2021; Goloborodko et al., 2016; Liao et al., 2025).

The minimal cell JCVI‐Syn3A offers an attractive system to study chromosome segregation in a simplified context. Chromosome conformation capture sequencing (3C‐seq) contact maps indicate that its chromosome is an unstructured fractal globule, with contact probabilities uniformly distributed across the genome except for high local contacts within 1 kb (Gilbert et al., 2021). Transcription‐induced supercoiling is not expected to form domains, consistent with these uniform contact patterns. JCVI‐Syn3A contains only a few DNA‐associated factors: the condensin homolog Smc–ScpAB, topoisomerases type I and II, and a low copy number of histone‐like nucleoid‐associated protein (HU) proteins (Gilbert et al., 2023). Cryo‐electron tomography (cryo‐ET) imaging of a representative small and large JCVI‐Syn3A cell further shows that the nucleoid, which fills the entire cell volume, is approximately uniformly interspersed with ribosomes (Gilbert et al., 2021), unlike the excluded‐ribosome nucleoids observed in *Escherichia coli* (Roberts et al., 2011).

Previously we introduced a computational framework to model the JCVI‐Syn3A chromosome (543 kbp) as an elastic wormlike chain at a resolution of 10 bp per bead, implementing the effects of SMC protein complexes, DNA replication, and topoisomerases (Gilbert et al., 2023). The model allows for simulation of excluded‐volume interactions between macromolecular complexes, such as ribosomes, with the chromosome. The computational framework is implemented in the program btree_chromo which calls large‐scale atomic/molecular massively parallel simulator (LAMMPS) as a library to perform energy minimizations and Brownian dynamics (BD). Recently we utilized this software in a 4D whole‐cell model (4DWCM) of the JCVI‐Syn3A cell cycle, with a few key modifications to the simulation procedure, including running LAMMPS on the graphics processing unit (GPU) instead of the central processing unit, and extending cell membrane (boundary particle) shapes to include overlapping spheres, which captures the prolate/dumbbell morphologies and roughly symmetric spherical daughter cells seen in cryo‐ET and fluorescence imaging. These changes are detailed in our 4DWCM study (Thornburg et al., 2026).

**FIGURE 1:**
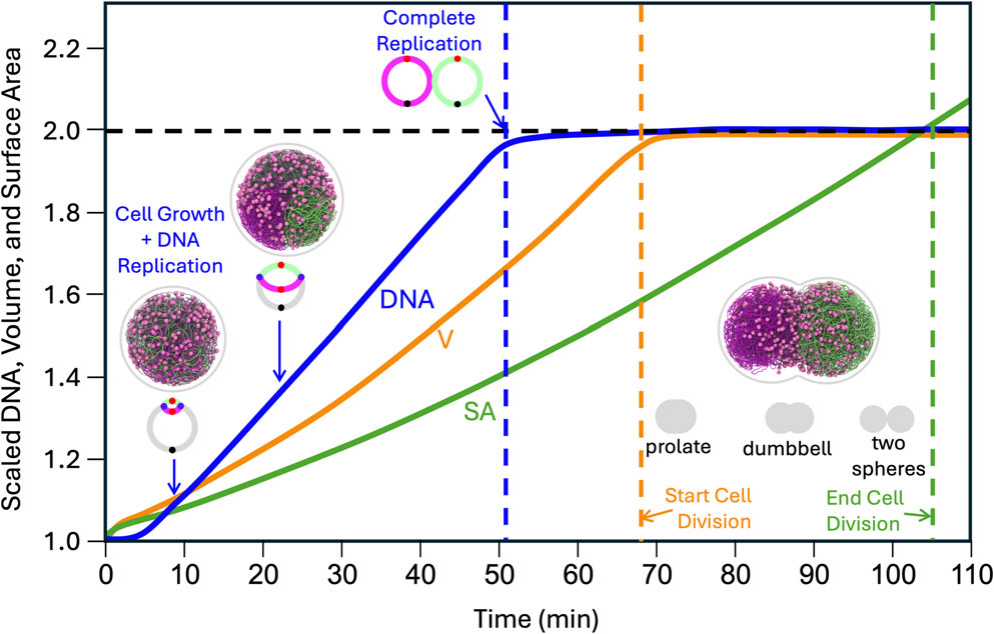
Cell geometry in relation to doubling times. DNA (blue curve) doubles in roughly 50 min. Volume (orange curve) doubles in roughly 65 min. Surface area (green curve) doubles in roughly 105 min. Snapshots of the cell at various points in the cell cycle are shown at 8, 22, and 80 min into the cell cycle. Cartoons of theta structures depicting replication states are shown, with daughter chromosomes in lime and magenta and mother chromosome in gray, and with the origin of replication, replication forks, and terminus shown as red, violet, and black dots. Between 65 and 105 min, the cell progresses from a sphere of radius 252 nm, to a prolate shape, to a dumbell shape, and finally to two spheres each with radius 200 nm.

A major result from the 4DWCM was that the simulated doubling times closely matched experimental measurements. A representative 4DWCM cell‐cycle trajectory (Figure 1) shows the following average timings: initiation of DNA replication at 5 min, completion of chromosome replication by 50 min, doubling of cell volume by 65 min, and doubling of cell surface area by 105 min. The replication rate used in our simulations (100 bp/s) is motivated by experimental measurements in *Mycoplasma* (Seto & Miyata, 1998). The simulated doubling time is nearly identical to the experimentally measured 105‐min doubling time. Moreover, these timings, when interpreted as representative of an unsynchronized population, yield an Ori:Ter ratio of 1.28—consistent with the experimentally observed ratio of approximately 1.2 from DNA sequencing of cells in mid‐ to late‐exponential phase (Thornburg et al., 2026).

Despite this strong agreement, one key limitation of the 4DWCM was its reliance on a small, ~12 pN fictitious force to drive segregation of the daughter chromosomes. Although effective, this mechanism is biologically artificial. We therefore asked whether spontaneous chromosome segregation could emerge in our model without the use of a fictitious force.

**FIGURE 2:**
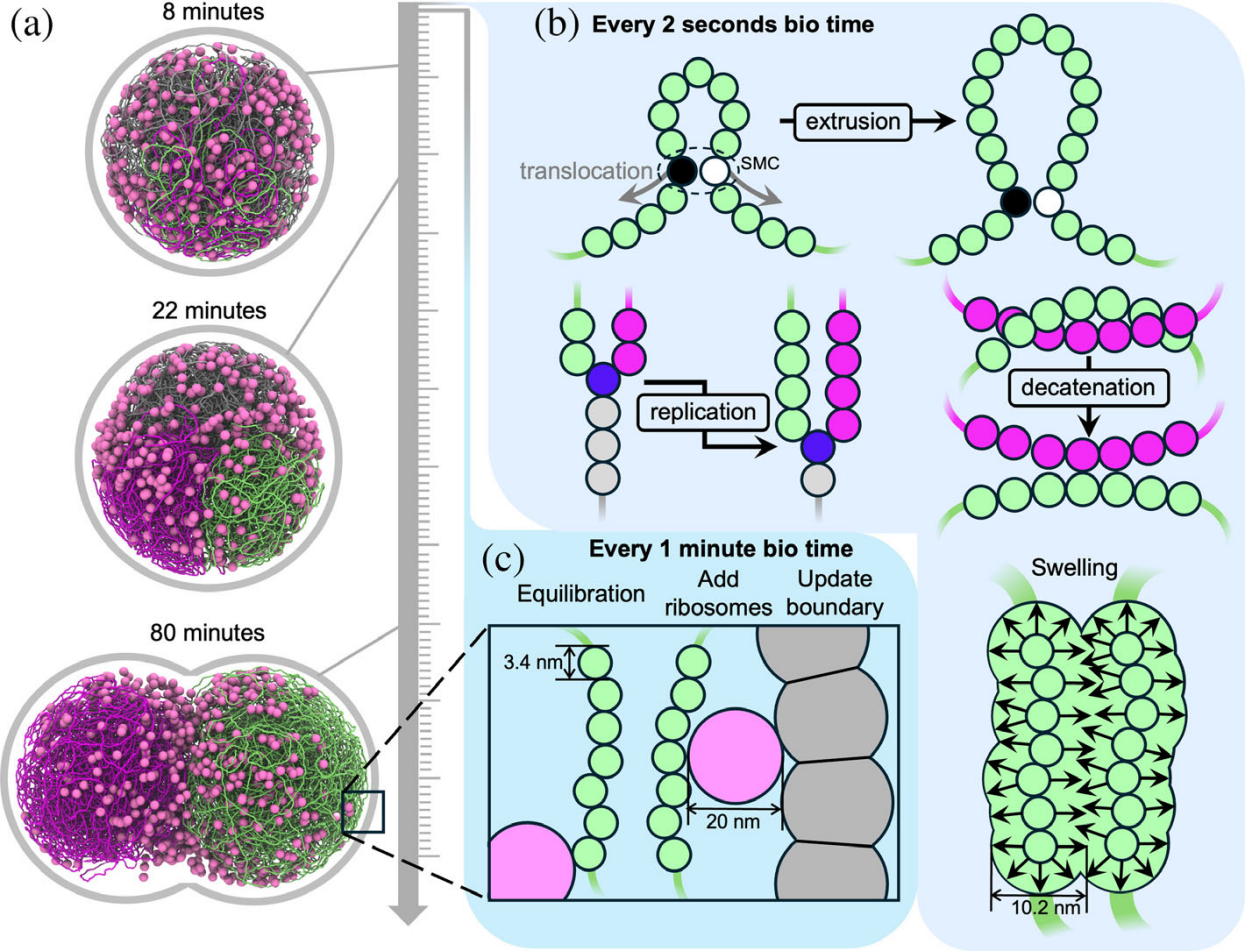
Simulation of JCVI‐Syn3A over the 105 min cell cycle. (a) Snapshots of cell at 8, 22, and 80 min into the 105 min cell cycle. (b) Every 2 s of biological time, the structural maintenance of chromosomes (SMC) positions and replication fork positions are updated. An energy minimization is performed, with strand crossing allowed to emulate the action of topoisomerases. An equilibration with an expanded excluded volume diameter for the DNA beads is performed in order to counteract the compaction caused by SMC. (c) Every 1 min of biological time, an extra‐long equilibration with the normal DNA diameter is performed. The coordinates for the contact maps are then sampled. Ribosomes are then added, and the boundary geometry is updated.

To address this question, we focused on the chromosome component of the JCVI‐Syn3A model, replacing the explicit handling of metabolism and other genetic information processing with simple growth curves for the model components. We introduced a relaxation procedure incorporating a “swelling” stage to counteract SMC‐induced compaction, and we updated the simulation framework to support tunable SMC number (N), translocation speed (v), and dwell times (τ), and explicit SMC–SMC and SMC–replisome stalling and bypassing interactions. The updated simulation workflow is summarized in Figure 2 and described in greater detail in Section 5. We found that a single parameter—loop coverage, defined here as the product Nvτ divided by the chromosome length L, and interpreted roughly as the expected fraction of the chromosome extruded into loops—is a strong predictor of chromosome partitioning (i.e., segregation) outcomes. However, loop coverage is not a perfect control parameter, that is, partitioning results are not totally decoupled from the individual parameters. We find that spontaneous segregation can occur provided sufficient loop coverage, whereas SMC–SMC blocking/bypassing dynamics and blocking/unloading of SMC due to collisions with the replication forks do not play a major role. Simulations without SMC–SMC blocking also show the best agreement with 3C‐seq DNA contact maps, although extremely large SMC processivities are required to recover the experimentally observed uniformity. Finally, we extend our framework to generate predictions across the entire cell cycle, providing testable hypotheses for future experiments using synchronized cell populations.

## 2 RESULTS

### 2.1 Maintaining an expanded JCVI‐Syn3A nucleoid during simulations of SMC action requires a “swelling” equilibration step

We noticed that in our previous 4DWCM simulations, successive loop extrusion steps would compact the chromosome, even when we permit strand crossings. Permitting strand crossings emulates the action of topoisomerases and is implemented by reducing the strength of the repulsive potential between DNA strands. The compacted chromosome conformations are in direct disagreement with cryo‐ET, which show an expanded nucleoid with an approximately uniform distribution of ribosomes (Gilbert et al., 2021). Furthermore, polymer theory predicts that the chromosome should be fully relaxed, based on the relatively fast relaxation rate of a loop of DNA compared to the extrusion rate of SMC (Chan & Rubinstein, 2023; Rubinstein & Colby, 2003). We found that nucleoid compaction could be partially alleviated through BD; however, the BD equilibrations performed for each 2‐s biological interval correspond to only microseconds of simulated time.

**FIGURE 3:**
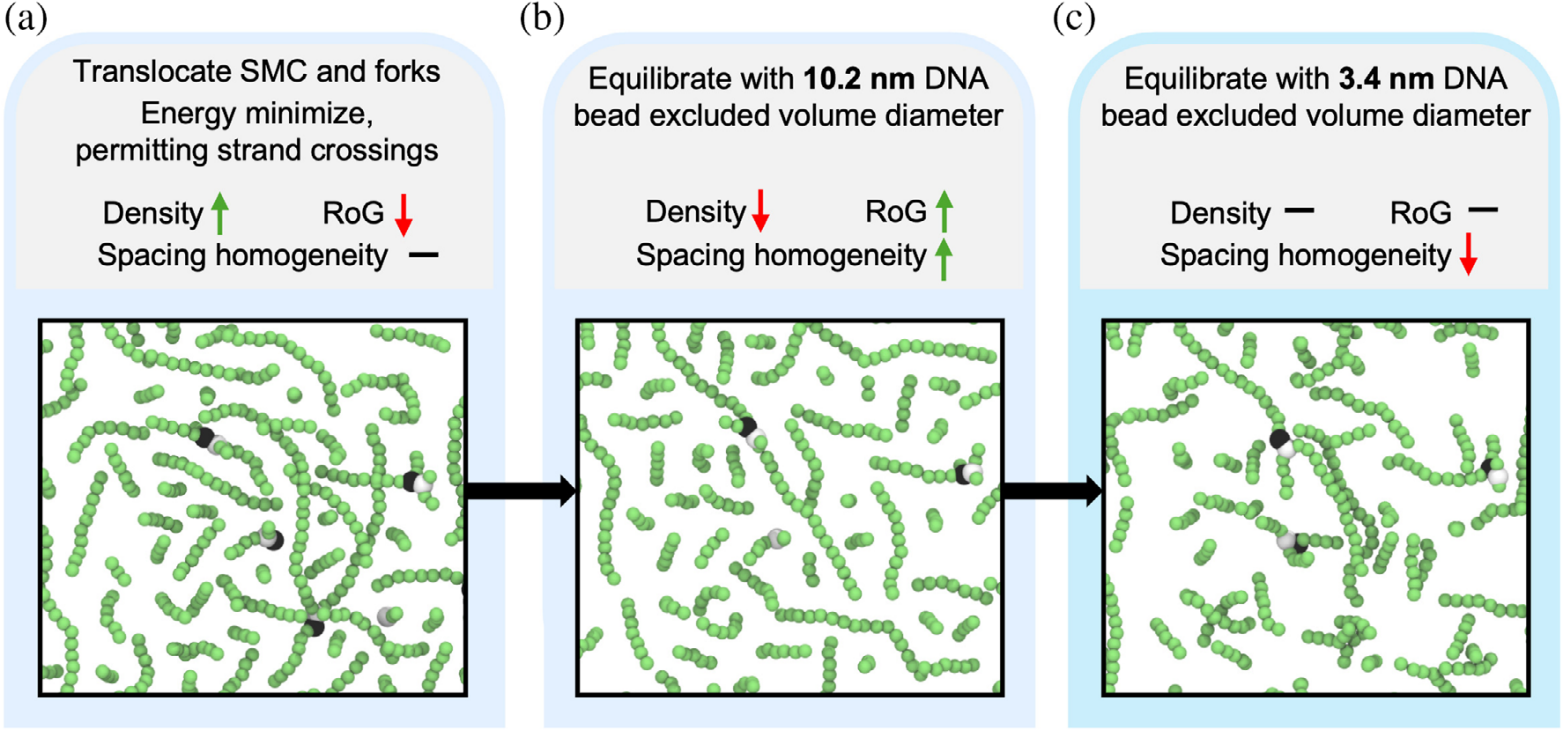
Swelling procedure. (a) Every 2 s of biological time, the structural maintenance of chromosomes (SMCs) translocate and DNA replication occurs. An energy minimization occurs with strand crossings permitted. The resulting density of the chromosome tends to increase. (b) In order to counteract this, following the energy minimization an equilibration is performed with DNA beads having a 10.2 nm excluded volume diameter. The density decreases and the radius of gyration (RoG) increases, but the spacing of the polymer network becomes excessively homogenous. (c) Every 1 min of biological time, a longer equilibration is performed with a 3.4 nm excluded volume diameter which restores the heterogeneity of the spacing between DNA segments. All renders are of a *z*‐slice of the DNA taken at the midplane of the cell, with a slice height of 12 nm.

In order to expand the chromosome, we introduce a relaxation step in which we equilibrate the system at a DNA bead diameter of 10.2 nm, which is three times the basal diameter of 3.4 nm. This procedure successfully increases the radius of gyration of the chromosome, but also produces two main side effects. First, it increases the apparent DNA persistence length to ~55 nm versus the basal ~44 nm due to the tendency of the swollen state to favor more extended chain conformations. Second, it renders the spacing between adjacent DNA segments more homogeneous (Figure 3b), yielding a mesh with a uniform pore size of 10.2 nm. To recover the persistence length of ~44 nm and inter‐strand spacing for DNA beads with diameter 3.4 nm, a long equilibration is performed every 1 min of biological time (Figure 3c) where DNA bead diameter is restored to 3.4 nm.

We argue that this procedure effectively recovers the equilibrium structure of the nucleoid. Although in principle, sufficiently long BD simulations would converge to similar chromosome conformations, such timescales are inaccessible in practice. Our swelling‐based relaxation step compensates for this limitation while preserving biological intent: by interleaving replication, SMC activity, and relaxation in discrete intervals, the model sequentially reconstructs processes that occur concurrently in the cell, rather than imposing an artificial behavior. This strategy offers a practical compromise between computational feasibility and more realistic cell biology with our present understanding.

On the other hand, it is also possible that simulating additional Brownian diffusion alone cannot expand the nucleoid to the degree observed in cryo‐ET. In that case, the swelling procedure may be compensating for inaccuracies or missing biophysical components in the model that would otherwise produce this level of expansion. Recent computational studies seem to corroborate this view: Roure et al. simulated a larger, 100 bp per bead chromosome model of JCVI‐Syn3A and showed that the nucleoid tended to compactify and expel ribosomes, an effect which was enhanced by nucleoid stiffness, HU concentration, and protein crowding, and counteracted by electrostatic interactions (Roure et al., 2025). These findings suggest that excessive nucleoid compaction may be a systematic limitation of the current generation of chromosome simulations.

If that is indeed the case, it is reasonable to hypothesize that electrostatic interactions could be the missing biophysical component that the increased excluded volume diameter is compensating for. Theory predicts that in 100 mM of monovalent salt, the effective width of DNA is approximately 5 nm (Frykholm et al., 2022; Stigter, 1977). That is bigger than the original 3.4 nm but half of the 10.2 nm used here, although we did not attempt swelling with a smaller diameter. This explanation is consistent with the study mentioned above which found that electrostatic repulsion promoted nucleoid expansion (Roure et al., 2025).

**FIGURE 4:**
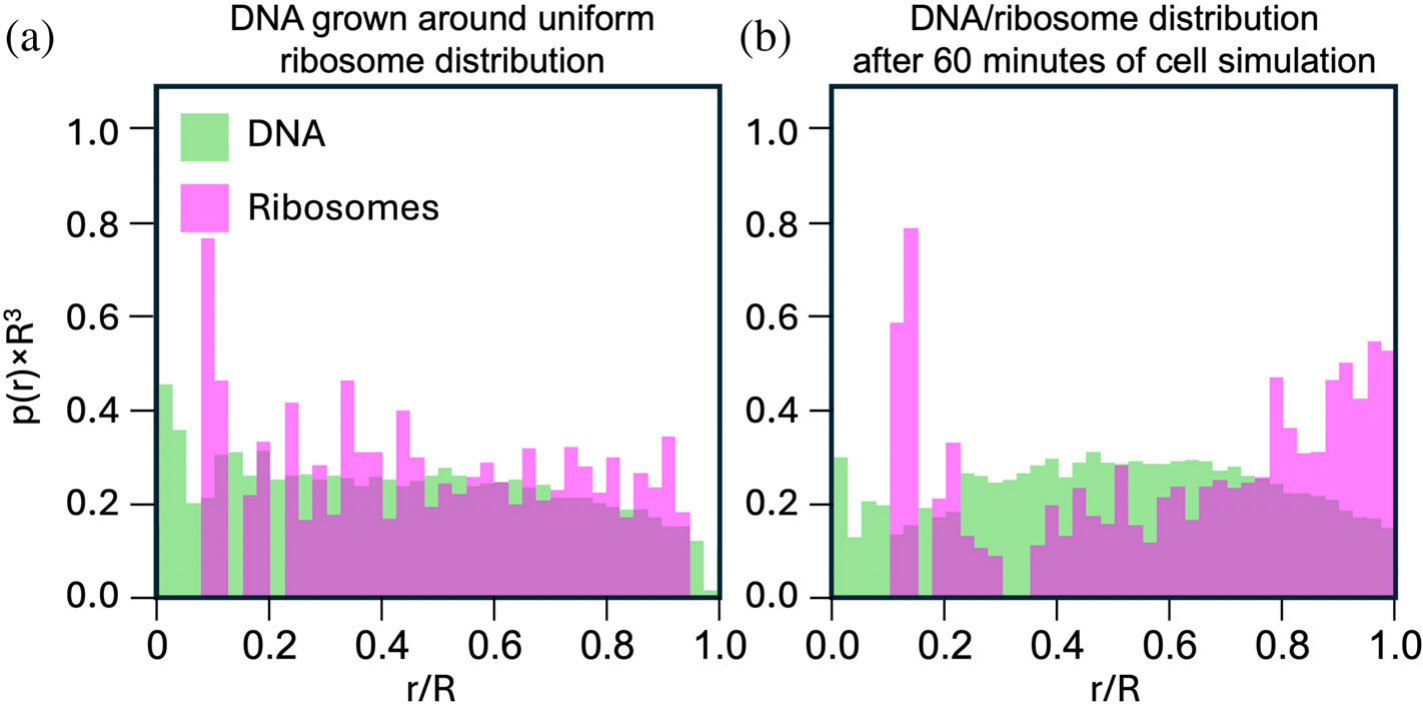
DNA and ribosomes distributions as a function of normalized radius r/R where R the cell radius. The y axis is probability per unit volume $p\left(r\right)$ of finding a DNA bead (lime) or ribosome (magenta) at normalized radius r/R, scaled by $R^{3}$ as to make the units on the y axis dimensionless. The parameters used were *N* = 50, *ν* = 500 bp/s, and *τ* = 75 s (loop coverage 1.38). (a) Prior to dynamics, the chromosome is initialized as a fractal globule threaded around a uniformly distributed ribosome field as observed from cryo‐electron tomography of a small JCVI‐Syn3A cell (Gilbert et al., 2021), using an iterative Koch‐curve algorithm. The cell radius is *R* = 206 nm. (b) After 60 min of cell cycle simulation (replication, structural maintenance of chromosomes looping, topoisomerases), ribosomes are displaced toward the cell periphery as the chromosome compacts. The cell radius is *R* = 252 nm.

We also observed ribosomes being pushed toward the cell periphery in our simulations, as illustrated in Figure 4. In Figure 4, DNA is compacted and ribosomes are partially expelled from the nucleoid even when using the swelling procedure in Figure 3. In our case this effect arises not only from excluded‐volume interactions, as in Roure et al. (2025), but also from SMC‐mediated DNA compaction. Furthermore, we believe that the swelling procedure also contributes to the expulsion of ribosomes, since the relatively homogeneous spacing between DNA strands produces a mesh‐like network with a pore size of roughly 10.2 nm, which is smaller than the diameter of ribosomes (~20 nm). Consequently, ribosome exclusion in our simulations occurs rapidly, typically within a few minutes.

We note that the mechanism for exclusion of ribosomes observed here differs from that in *E. coli*, where ribosome exclusion from the nucleoid and accumulation in the center of the cell in between daughter chromosomes has been suggested to facilitate chromosome segregation cells across different nutrient conditions and growth rates (Papagiannakis et al., 2025). In particular, Papagiannakis et al. attribute ribosome accumulation between segregating nucleoids to nonequilibrium translation–diffusion dynamics of polysomes over extended timescales, which generates steric pressure that promotes segregation. In our simulations, ribosome exclusion to the periphery happens rather quickly, and at 60 min after initiation of DNA replication we do not observe significant accumulation of ribosomes between daughter chromosomes. Consistent with this difference in mechanism, ribosome exclusion does not appear to contribute to chromosome segregation in our model. Polysomes are not included in the present simulations, although experimental measurements suggest that roughly 20%–40% of ribosomes in Syn3A could participate in polysomes based on a distance metric (Gilbert et al., 2021). However, without knowing the orientation of the ribosomes, one cannot estimate the degree of polysome formation.

We also saw ribosome exclusion happening in the 4DWCM (Thornburg et al., 2026), but we did observe a slight accumulation in between daughter chromosomes as space becomes available. However, there the diffusion of the ribosomes is simulated over a longer time, and a fictitious force to separate the chromosome is used. We additionally observe that ribosome exclusion becomes more pronounced in simulations with higher loop coverage, and that simulations without the swelling procedure, which produce more strongly compacted chromosomes, exclude ribosomes even more strongly. Overall, the mechanisms governing ribosome exclusion in our model remain incompletely understood and warrant further investigation.

Finally, we note that our strategy of using a large bead size to facilitate chromosome segregation is reminiscent of the model of Goloborodko et al., which simulated sister chromatid separation and disentanglement (Goloborodko et al., 2016). Notably, both approaches employ similar bead sizes and system scales: Goloborodko et al. used 10 nm beads with 50,000 total beads, while our model uses a swollen bead size of 10.2 nm with 54,338 total beads (when unreplicated). In their case, each bead corresponds to three nucleosomes (600 bp), with a total modeled chromosome size of 30 Mb, while here each bead represents 10 bp with a total chromosome size of 543 kb.

### 2.2 Loop coverage predicts partitioning outcomes

**FIGURE 5:**
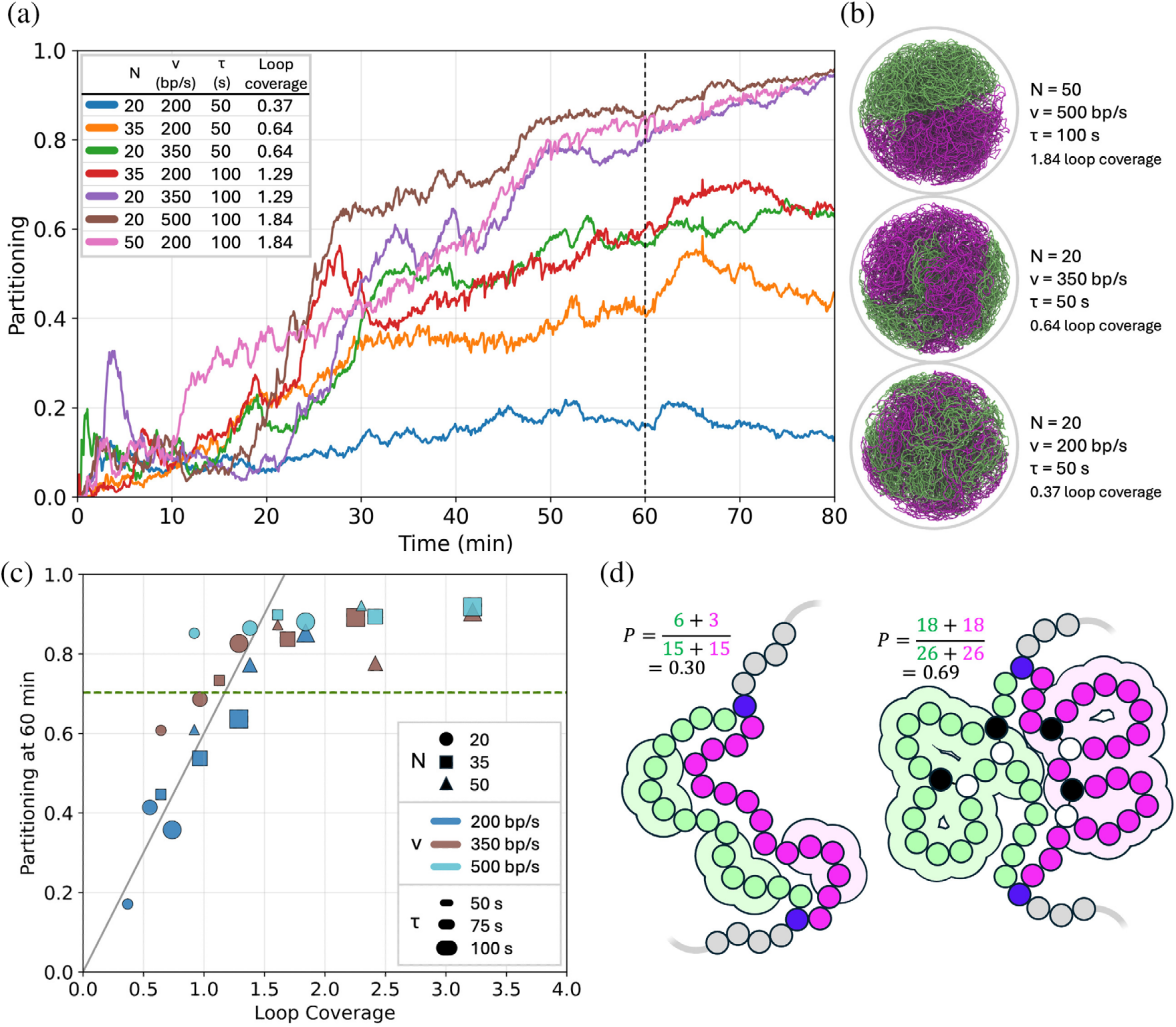
Partitioning of chromosomes for cell cycles simulated with different numbers of active structural maintenance of chromosomes (SMC) per chromosome (N), SMC translocation speed (v), and SMC dwell times (τ). (a) Partitioning of daughter chromosomes for 80 min of the cell cycle following replication initiation. Replication finishes at ~45 min, and cell division begins at 60 min (black dashed line). (b) Snapshots of three representative simulations from the previous panel taken at 60 min. (c) Partitioning of chromosomes at 60 min plotted against loop coverage, defined as Nvτ/L where L is the chromosome size. Each point corresponds to a simulation performed using a unique parameter set, with N indicated by shape, v by color, and τ by size. All simulations for which partitioning exceeded 0.7 at 60 min (points above green dashed line) continued on to fully partition by the end of the cell cycle. (d) Cartoon illustration of the method used to calculate partitioning. A daughter bead is considered partitioned if no beads from the opposite daughter are found within a 30 nm radius (indicated by the lighter‐colored bubble around the bead). Partitioning is defined as the fraction of daughter beads meeting this criterion.

To examine how chromosome segregation depends on SMC parameters, we performed a systematic parameter scan across the number of SMC complexes N, the translocation speed v, and the dwell time τ. Specifically, we simulated three values of each parameter (N=20,35,50; v=200,350,500bp/s; τ=50,75,100s). Partitioning of the daughter chromosomes during the 80 min following replication initiation is shown in Figure 5a. Across this parameter space we found that partitioning outcomes correlate strongly with the *loop coverage*, defined as Nvτ/L, which can be roughly interpreted as the expected fraction of the chromosome extruded into loops (here *L* = 543,379 bp). The quantity Nvτ/L has been discussed in previous simulation studies of SMC‐driven chromosome organization, where it has been shown to correlate with both linear lengthwise chromosome compaction and sister chromatid separation in eukaryotes (Goloborodko et al., 2016), as well as positioning of chromosome arms in *E. coli* (Harju et al., 2025). Parameter sets with similar loop coverage generally produced similar partitioning dynamics. Representative snapshots of cells at 60 min after the start of DNA replication are shown in Figure 5b. We note that although simulations with large loop coverage (e.g., N=50, v=500bp/s, τ=100s) appear visually fully partitioned at this time point, contacts between daughter chromosomes persist near the chromosome boundaries until the cell membrane fully divides into two spheres at approximately 105 min. As shown in Figure 5c, the degree of partitioning at 60 min is well predicted by loop coverage, with partitioning increasing roughly proportionally with Nvτ/L until reaching a saturation level determined by the geometric constraints of the dividing cell. In practice, all simulations that reached a partitioning value of 0.7 by 60 min went on to complete chromosome segregation by the end of the cell cycle, whereas simulations below this threshold failed to fully partition. In general simulations with loop coverages >1 partitioned, although there were two outliers: N=20, v=500bp/s, τ=50s, corresponding to loop coverage 0.92, partitioned, while N=35, v=200bp/s, τ=100s, corresponding to loop coverage 1.29 (red trace in Figure 5a), did not. The method used to calculate partitioning is illustrated schematically in Figure 5d.

Our results explain why a fictitious partitioning force was required to segregate chromosomes in earlier versions of the model (Thornburg et al., 2026). In those simulations, we assumed ~50 actively translocating SMC per chromosome and a 500 bp/s translocation speed, but employed a short dwell time of 4 s corresponding to the hook interval in our hybrid simulations. This corresponds to a loop coverage Nvτ/L of 0.18, which is far below the value of 1 that we have found is the cutoff for reliable segregation.

**FIGURE 6:**
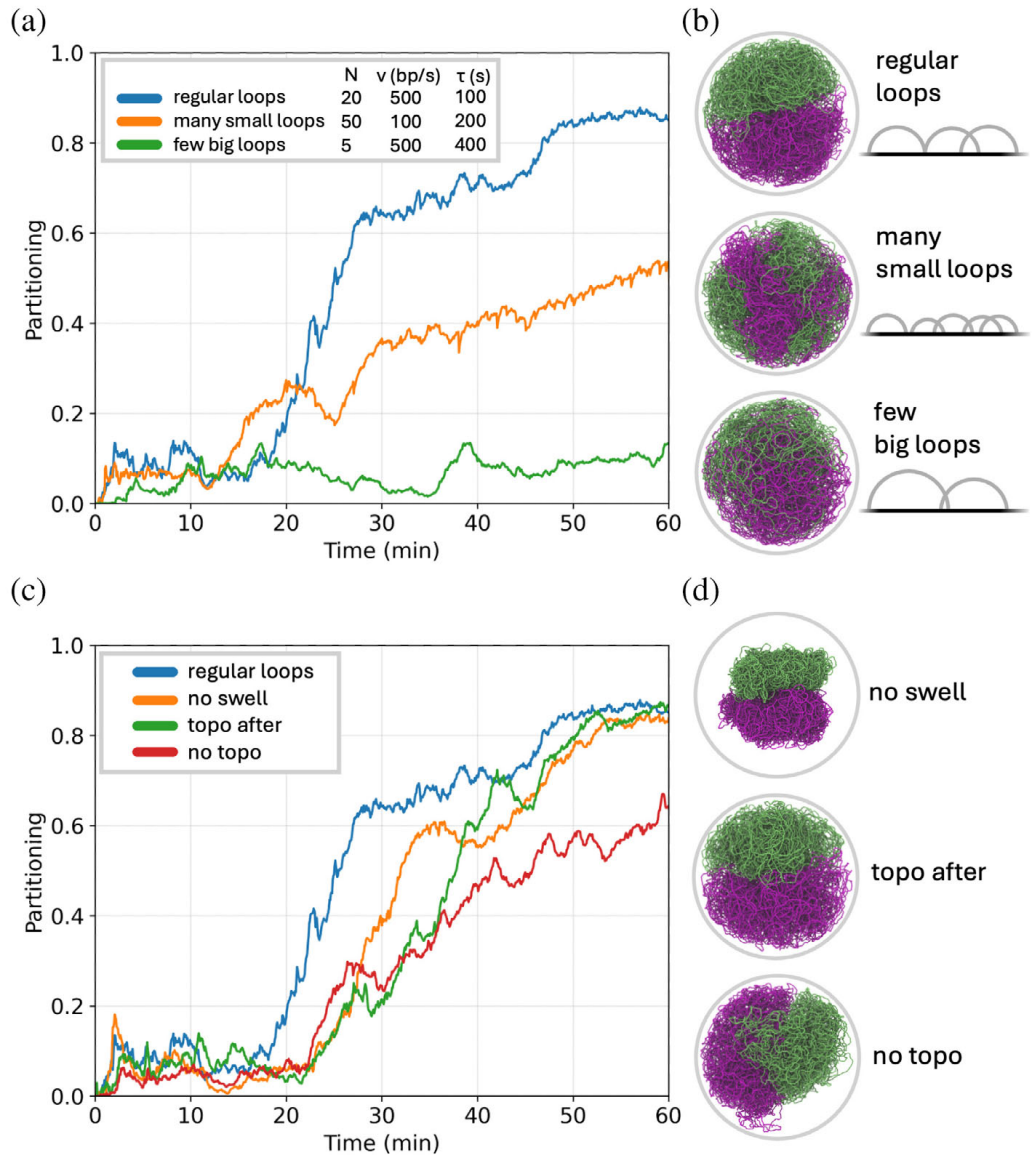
Partitioning of chromosomes for cell cycles simulated under different conditions. (a) Partitioning of daughter chromosomes for “regular loops” with *N* = 20, *ν* = 500 bp/s, and *τ* = 100 s; “many small loops” with *N* = 50, *ν* = 100 bp/s, and *τ* = 200 s; and “few big loops” with *N* = 5, *ν* = 500 bp/s, and *τ* = 400 s. In all three cases the loop coverage Nvτ/L=1.84. (b) Snapshots of the cell corresponding to the parameter sets from the previous panel, 60 min after the start of DNA replication. (c) Partitioning of daughter chromosomes for simulations without swelling (orange), topoisomerase action after structural maintenance of chromosomes loop extrusion rather than concurrent (green), and without topoisomerase action (red). Simulations used the same N, v and τ as the “regular loops” parameter set from the previous panel, included as well for comparison (blue). (d) Snapshots of the cell corresponding to the parameter sets from the previous panel, 60 min after the start of DNA replication.

Although loop coverage provides a useful predictor of segregation outcomes, it is not a perfect control parameter. To test the limits of this scaling, we compared parameter sets with identical loop coverage but different combinations of N, v, and τ (Figure 6a). Starting from a parameter set that reliably partitions chromosomes (N=20, v=500bp/s, τ=100s), we constructed two extreme cases while keeping the loop coverage fixed at Nvτ/L=1.84. In one regime (“many small loops”), we increased the number of SMC complexes (N=50) while reducing processivity (v=100bp/s, τ=200s). In the opposite regime (“few big loops”), we considered a small number of highly processive SMC complexes (N=5, v=500bp/s, τ=400s). Despite having identical loop coverage, both extreme regimes exhibited slower partitioning dynamics than the reference parameter set, and segregation did not complete within the simulated cell cycle. These results indicate that although loop coverage captures the dominant scaling of segregation behavior, efficient partitioning occurs only within a favorable region of parameter space. Similar optimal loop‐size effects have been reported in previous polymer simulations of loop extrusion (Brahmachari & Marko, 2019).

Finally, we tested whether several modeling choices influenced the segregation mechanism. First, we examined the role of the swelling equilibration step used to maintain an expanded nucleoid configuration during simulations with active SMC complexes. Simulations performed without this swelling step still exhibited chromosome partitioning when loop coverage was sufficiently large (e.g., N=20, v=500bp/s, τ=100s, loop coverage 1.84), indicating that swelling is not strictly required for segregation in the model (“no swell” in Figure 6c,d). A scan over the same set of parameters described above was performed for simulations without swelling, and partitioning exhibited the similar dependence on loop coverage. We also tested whether the ordering of SMC loop extrusion and topoisomerase‐mediated strand crossings affected the results. In additional simulations, topoisomerase minimizations were performed after the SMC updates rather than concurrently with them. Under conditions of sufficient loop coverage, partitioning outcomes were unchanged (“topo after” in Figure 6c,d), suggesting that the segregation mechanism does not depend sensitively on the precise ordering of these processes. In contrast, simulations performed without topoisomerase‐mediated strand crossings failed to achieve full chromosome segregation, demonstrating that strand‐passing activity is required for efficient partitioning in our model (“no topo” in Figure 6c,d).

### 2.3 SMC–SMC blocking and bypassing dynamics and replisome facilitated unloading have minimal impact on determining if chromosome segregation occurs

Although bypassing replication forks is not included in the current model, evidence from Hi‐C maps of *B. subtilis* cells with synchronized DNA replication indicates that fork bypass occurs at a nonzero frequency (Liao et al., 2025), and blocking of SMC by the replisome was observed in single molecule experiments (Glaser et al., 2025). In our previous work, looping was considered only within like daughters/mother chromosomes, but a “grab radius” allowed SMCs to randomly interact with candidate segments (Gilbert et al., 2023). Here, we retain the assumption that looping past replication forks is not allowed and do not permit inter‐strand grabbing, although recent simulation/experimental studies indicate that such events indeed happen occasionally (Bonato et al., 2025). Furthermore, while stalling between SMC and RNA polymerase (RNAP) has been observed, translocating SMC have been shown to bypass transcribing RNAP within 1–10 s in *B. subtilis* (Brandão et al., 2019). Transcription and supercoiling are not considered in the present model and RNAP are not present in the model.

**FIGURE 7:**
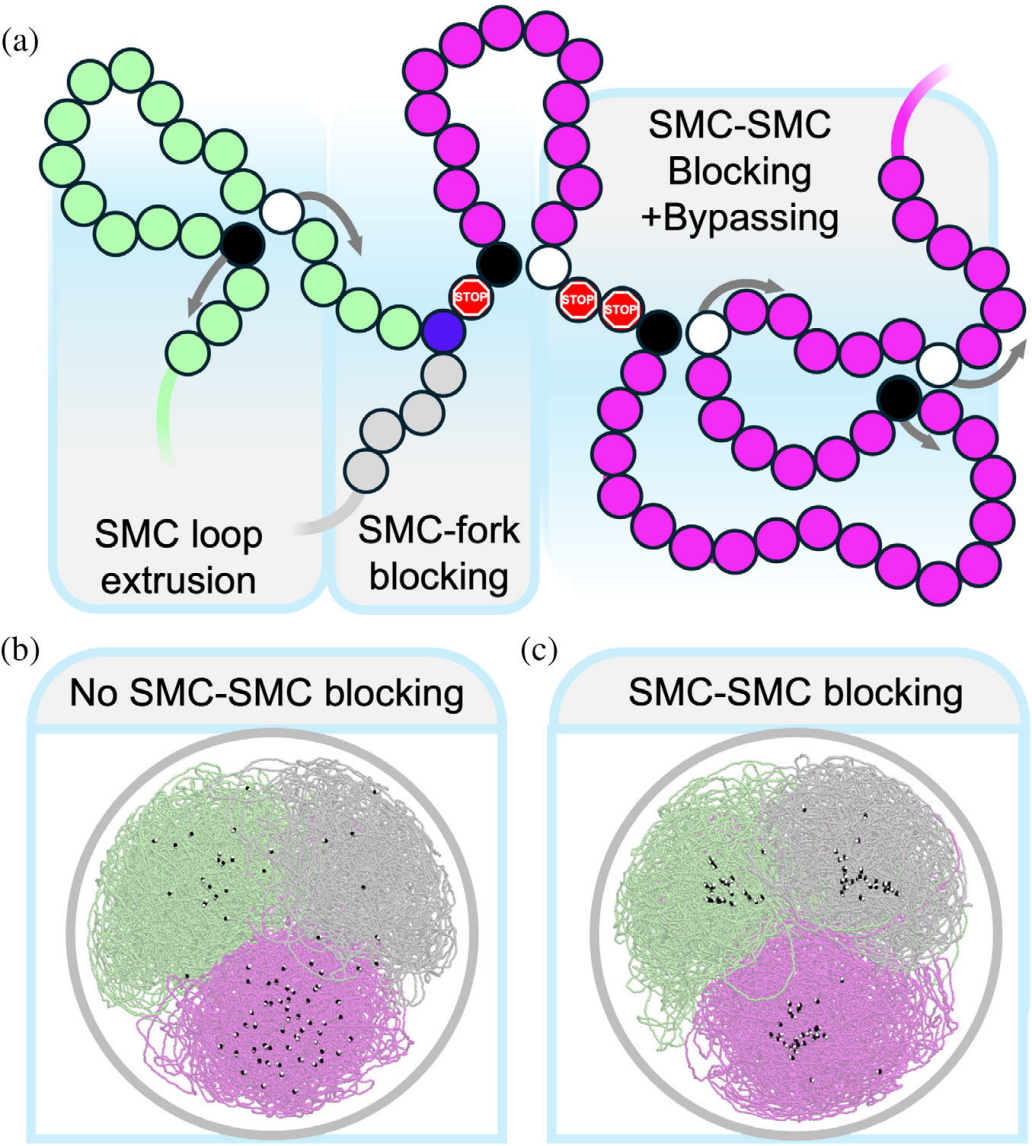
Structural maintenance of chromosomes (SMC)–SMC blocking and bypassing. (a) Schematic representation of SMC loop extrusion, blocking of SMC extrusion by replication fork, and blocking/bypassing between two SMC. SMC are taken to have an effective width of three DNA beads. Red beads indicate DNA beads that are “off limits” for SMC extrusion. (b) Removing the possibility of SMC–SMC blocking results in uniform SMC disribution within daughter (lime and magenta) and mother (gray) chromosome territories. (c) Blocking between SMC results in clustered SMC distribution within each chromosome territory. Black/white beads represent left/right sides of SMC complexes.

Real‐time imaging of yeast condensin on flow‐stretched DNA indicates that SMCs translocating toward one another can block and then bypass each other within seconds (Kim et al., 2020). However, like the SMC dwell time, the SMC–SMC blocking time in vivo remains poorly characterized. Simulations reproduce Hi‐C maps of ParS‐site engineered *B. subtilis* strains most accurately when the SMC–SMC blocking time is set to 20 s (Brandão et al., 2021). We extended our looping model to include both blocking and bypassing between SMCs (Figure 7). Simulations testing blocking/no blocking used *N* = 50, *ν* = 500 bp/s, and *τ* = 100 s. For simulations with SMC–SMC blocking enabled, we used a blocking time of 20 s as inferred from *B. subtilis* Hi‐C data (Brandão et al., 2021). SMC are taken to have a width of 3 DNA beads on the chromosome, that is, two SMC can translocate until they are 3 beads away from one another, at which point they are considered blocked.

Turning the SMC–SMC blocking dynamics on did not prevent partitioning from occurring. However, SMCs become clustered near the center (Figure 7c). The clustering is expected for high loop coverages. Since extrusion is double‐sided, once a jam occurs between two SMCs, an existing free side of the two SMCs will continue to translocate until they reach another SMC/SMC jam, a process which occurs repeatedly to form clusters. There is evidence from single molecule tracking of Smc–ScpAB in *B. subtilis* that SMC protein complexes form several small clusters in vivo, which contain two to three SMC protein complexes, more similar to Figure 7b than the large clusters in Figure 7c (Schibany et al., 2018).

In our simulations, we assume that SMC complexes approaching the replication fork from the mother side are displaced by the incoming replication fork. SMCs that catch up to the replication fork from the daughter side will stall and follow behind the replication fork until they eventually unbind. We note that in our simulations, SMCs have a fast translocation rate (200–500 bp/s) relative to the replication forks (100 bp/s), so many of them encounter one or both replication forks before unbinding. We tested whether localization of SMC complexes at the replication fork affects chromosome segregation by simulating scenarios in which SMC complexes immediately dissociate at forks when they catch up to them, or rapidly with an enhanced unbinding frequency (1 per 25 s, as reported by Liao et al., 2025), and compared them to simulations without modified unloading at replication forks (all tests with *N* = 20, *ν* = 500 bp/s, and *τ* = 100 s). In cases with immediate unloading, enhanced unloading, and no enhanced unloading, the overall segregation outcomes were similar, suggesting that replication‐fork localization is not a primary determinant of segregation in our model.

### 2.4 Simulations with high SMC processivity and without SMC–SMC blocking best reproduce 3C‐seq DNA contact maps

Previously we generated ensembles of DNA configurations constrained by ribosome positions obtained from cryo‐ET by growing a lattice polymer DNA model as a self‐avoiding polygon and compared the contact maps from these configurations to a contact map from 3C‐seq of cells in stationary phase (Gilbert et al., 2021). Later we generated contact maps based on a polymer model, in which a circular chromosome was grown using a Koch‐curve algorithm around a uniform ribosome distribution and shown to have a fractal globule structure (Gilbert et al., 2023).

**FIGURE 8:**
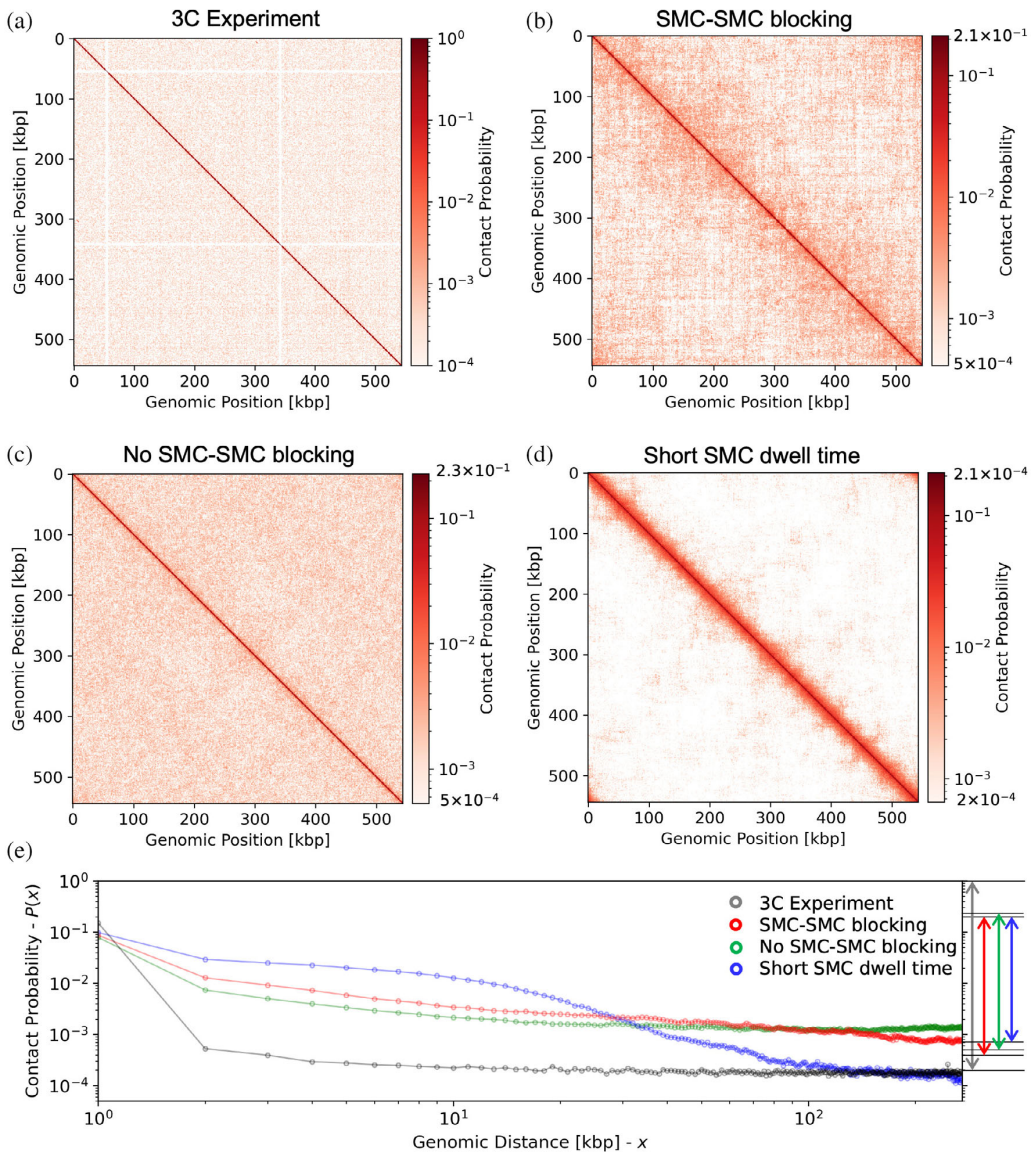
In silico contact maps of the JCVI‐Syn3A chromosome in stationary phase compared to 3C‐seq data at 1 kb resolution. The contact map from the 3C experiment is shown in panel (a). Simulations include structural maintenance of chromosomes (SMC)‐mediated loop extrusion under three conditions: (b) SMC–SMC blocking, (c) no SMC–SMC blocking, and (d) short SMC dwell time. Contact probability curves (e) show that the “no SMC–SMC blocking” condition best reproduces the experimental contact probability, including the plateau for distances >10 kb. The ranges used for the colorbars in (a–d) are depicted as colored arrows on the right side of (e).

Here, we generated contact maps for simulated cells in stationary phase, incorporating the effects of SMC‐mediated looping, and compared them to 3C‐seq maps at 1 kb resolution (Figure 8a). Because the cells are in stationary phase, we simulated looping only, without replication or cell growth (single chromosome, cell radius fixed at 200 nm, number of ribosomes fixed at 500). We considered three conditions: “SMC–SMC blocking,” “No SMC–SMC blocking,” and “Short SMC dwell time” (Figure 7b–d). The “SMC–SMC blocking” and “No SMC–SMC blocking” simulations employ a large dwell time of 2500 s, while the “Short SMC dwell time” employs a short dwell time of 4 s. Both simulations used *N* = 50 and *ν* = 500 bp/s. While the parameters chosen are unrealistic, they demonstrate the limiting behavior of contact probabilities for both large and small average loop sizes. For each condition, we ran 10 replicates of 10 min each. Coordinates were sampled at 1 min intervals (with the first sample taken after 1 min of dynamics) and summed across replicates and time points, yielding a total of 100 conformations per condition.

The 3C‐seq contact map has no domain structure (Figure 8a). Plotting the contact probability versus genomic distance (gray points, Figure 8e) reveals a relatively high contact probability for loci separated by 1 kbp, and a plateau in contact probability for distances greater than 10 kbp.

Of the three simulated conditions, the “no SMC–SMC blocking” scenario agrees best with the experimental data qualitatively, showing a relatively uniform contact distribution. The slope of the contact probability (green points, Figure 8e) for distances between 2 and 10 kb closely matches that of the 3C‐seq contact map, and it exhibits a similar plateau for distances beyond 10 kb. The “SMC–SMC blocking” (Figure 8b) and “short SMC dwell time” (Figure 8d) scenarios produce contact maps with apparent structure; however, this is largely due to the limited number of replicates. The “SMC–SMC blocking” scenario shows a similar overall trend to the no‐blocking case, but contact frequencies are elevated for distances of 1–100 kbp and reduced for 100–271 kbp. The “short SMC dwell time” scenario exhibits the weakest agreement with experiment at short distances (1–100 kb) but appears to match better at longer distances (100–271 kb); the agreement for longer distances likely arises as an artifact of the normalization procedure rather than reflecting true biological organization. The thick main diagonal with relatively fewer contacts at greater distances exhibited for the “short dwell time scenario” was also observed for the parameter sets in Section 2.2, with the extent of the drop‐off in contact probability correlated with the loop processivity vτ. The biological implications of these results are discussed in Section 3 below.

### 2.5 Simulations generate contact map predictions across the JCVI‐Syn3A cell cycle

Previously, we developed a binary tree formalism to describe the replication state of the circular JCVI‐Syn3A chromosome and used it to generate in silico contact maps for chromosomes in a wide range of replication configurations, including nested theta structures (Gilbert et al., 2023). In contrast to that static framework, the present model predicts chromosome conformation dynamically across the entire cell cycle. Guided by the results of the preceding sections, we performed 10 production simulations with the relaxation procedure described in Section 2.1, *N* = 50, *ν* = 500 bp/s, *τ* = 100 s, and no SMC–SMC blocking. For each trajectory, we sampled the three‐dimensional (3D) coordinates at 1‐min intervals immediately following the long equilibration step (Figures 2c and 3c) and constructed contact maps from these sampled conformations.

**FIGURE 9:**
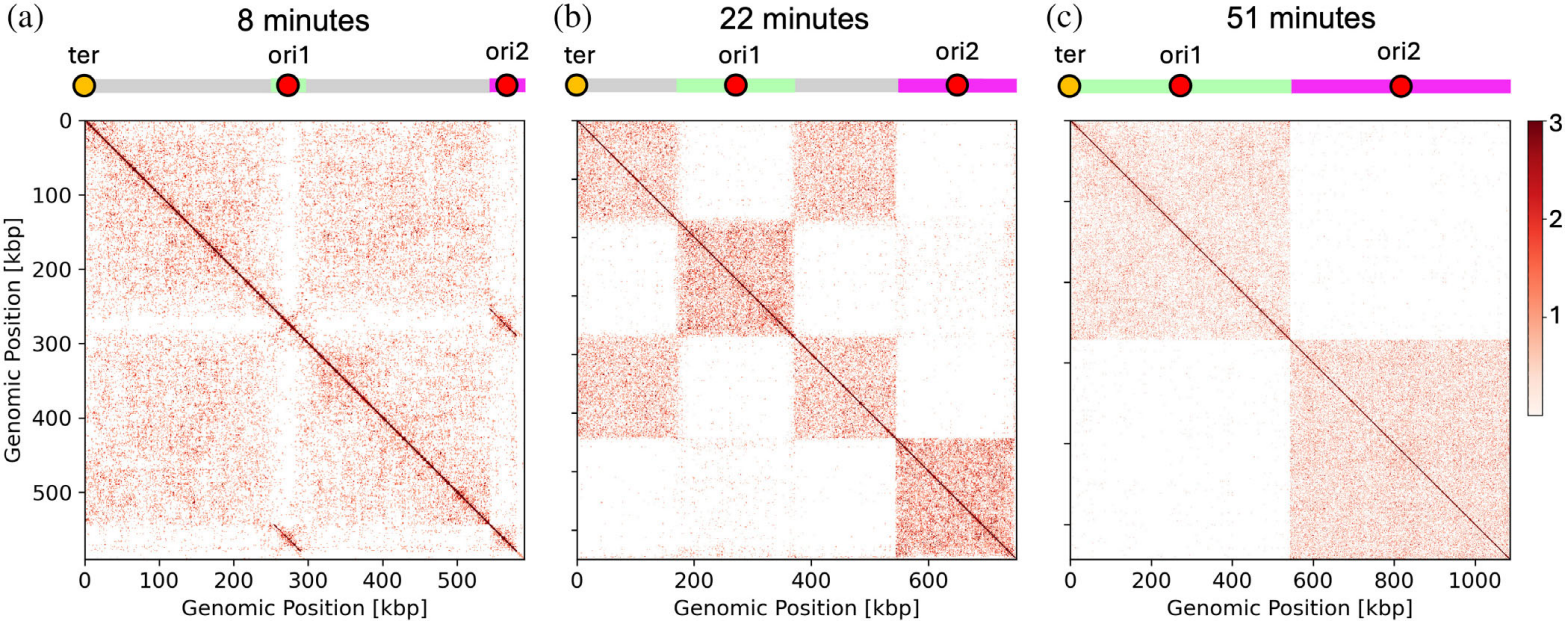
Dynamic in silico contact maps of the JCVI‐Syn3A chromosome across the cell cycle. A colored line shows the genomic positions corresponding to daughter chromosomes (lime and magenta) and mother chromosome (gray), with positions of the terminus (orange circle) and origins of replication of both daughter chromosomes (ori1 and ori2, red circles) indicated. Genomic positions of the left daughter chromosome are taken to correspond to the (previous) positions of the mother, with origin at *L*/2 = 271,689, while genomic positions of the right daughter are appended as extra indices (>543,379). Coordinates were sampled at 1‐min intervals from simulations with swelling enabled, *N* = 50, *ν* = 500 bp/s, *τ* = 100, and no structural maintenance of chromosomes (SMC)–SMC blocking. Because of the low number of counts, the color scale is capped at a maximum of three contacts. Three periods are evident: 0–5 min after the start of the cell cycle, single unreplicated chromosome; (a) 5–12 min, contacts between newly replicated daughter chromosomes appear as off‐diagonal blocks; (b) 12–50 min, DNA replication and SMC looping establish intra‐chromosome contact domains; (c) 50–105 min, replication completes and contact frequency decreases as chromosomes segregate and the cell grows.

The contact maps can be divided into three qualitatively distinct periods over the cell cycle. From replication initiation (5 min) to roughly 12 min (Figure 9a), contacts between the newly created daughters are visible as distinct diagonal features separate from the “main” diagonal (in Figure 9a, the contacts between the two different daughters, with genomic positions annotated as lime and magenta). In our simulations, this is due to the fact that SMC, which would be responsible for disentangling and segregating the daughters, has not had adequate time to bind and loop the daughters. From roughly 12 to 50 min (Figure 9b), DNA replication and SMC looping proceed and distinct regions are formed that correspond to intra‐chromosome contacts (mother–mother, like daughter with like daughter). Inter‐chromosome contacts are rare but are relatively more frequent between daughters than between daughter and mother, and exhibit higher frequency near the replication forks, due to SMC's stalling there. From 50 to 105 min (Figure 9c), replication has completed and the contact maps reflect growth of and segregation of the cells: as the cell volume increases, contacts decrease, and as segregation occurs, contacts between daughters, which were already sparse, trend to zero.

These in silico maps could be compared to 3C‐seq maps from synchronized cell populations. It should be noted that equivalent loci on daughter chromosomes are indistinguishable to 3C. However, features as seen in Figure 9 might still be resolvable: for example, the increased contact frequency of daughter–daughter contacts in Figure 9a would appear as increased contact frequency on the diagonal near the origin. More generally, the 3C‐seq maps from synchronized cell populations could serve as a constraint for inferring the SMC parameters that best reproduce the experimental data.

## 3 DISCUSSION

The parameter scan presented above shows that chromosome segregation in our simulations depends primarily on the loop coverage, defined as Nvτ/L. Successful partitioning occurs when loop extrusion generates sufficient coverage of the chromosome. A key question, however, is whether the parameter values required to achieve this regime are consistent with experimental measurements of SMC abundance and dynamics. In the following paragraphs we compare the ranges of *N*, *ν*, and *τ* that produce segregation in our simulations with available experimental estimates, and discuss how these constraints shape the physical interpretation of the mechanism proposed here.

The range of SMC copy numbers explored in our parameter scan (*N* = 20–50) is broadly consistent with estimates from proteomics experiments. Two datasets are currently available for JCVI‐Syn3A. The earlier dataset (Breuer et al. 2019) reports approximately SMC ~202, ScpA ~1, and ScpB ~31 copies, while a more recent dataset reports SMC ~48, ScpA ~8, and ScpB ~50 (Brier et al. 2025). The stoichiometry of the Smc/ScpA/ScpB complex is often assumed to be 2:1:2, although both in vitro (Rumyantseva et al., 2025) and in vivo (Schibany et al., 2018) studies suggest that this ratio may vary. Because of these uncertainties, and the inconsistent ScpA and ScpB abundances across datasets, the number of active SMC complexes cannot be determined unambiguously from proteomics measurements. We therefore estimate the maximum possible number of SMC complexes based solely on total SMC abundance. Assuming that SMC functions as a dimer, these measurements correspond to upper bounds of roughly ~100 and ~25 potential SMC complexes, respectively. Proteomics measurements also do not determine what fraction of complexes are DNA‐bound; single‐molecule tracking experiments in *E. coli* suggest that roughly half of MukBEF complexes are chromosome‐associated at any given time (Mäkelä and Sherratt 2020). If a similar fraction applies in JCVI‐Syn3A, the number of active SMC complexes would be ~12 to 50 per chromosome, consistent with the parameter range explored in our simulations. Simulations with too few active SMC complexes per chromosome were unable to partition the daughter chromosomes even with relatively large translocation speeds and dwell times.

Experimental estimates of SMC residence times remain uncertain but generally fall on the order of minutes. Single‐molecule tracking measurements indicate that MukBEF complexes in *E. coli* remain bound to the chromosome for a mean dwell time of ~65 s (Mäkelä and Sherratt 2020), while fluorescence recovery after photobleaching (FRAP) experiments suggest that SMC complexes in *B. subtilis* exchange between chromosome‐associated clusters and the surrounding pool on a similar minute timescale, with a recovery half‐time of ~2 to 3 min (Kleine Borgmann et al. 2013). In our simulations, dwell times in the range of 50–100 s were sufficient to support segregation provided that the number of active SMC complexes and the translocation speed produced adequate loop coverage. For example, simulations with $\left(N,v,\tau \right)=\left(50,200\;\mathrm{bp}/s,50\;s\right)$ did not result in partitioning, whereas (35, 350 bp/s, 50 s) did, despite the identical dwell time. These results illustrate that segregation outcomes depend primarily on the resulting loop coverage rather than on the dwell time alone.

Experimental estimates of SMC translocation speeds span roughly an order of magnitude across systems. In vitro experiments (Ganji et al., 2018) and in silico studies (Bonato et al., 2025) of yeast condensin report loop extrusion rates of ~0.1 to 1 kbp/s. These values are consistent with experimentally inferred translocation speeds of ~170 bp/s for *Gallus gallus* condensin and ~1 kbp/s for *B. subtilis* SMC complexes (Hassler et al., 2018). In *B. subtilis* growing at 42°C, the inferred SMC translocation speed of ~1.2 kbp/s closely matches the replication fork speed of ~1.1 kbp/s (Liao et al., 2025), consistent with observations that SMC translocation rates and replisome speeds are often similar across bacterial species. In our simulations, however, translocation speeds comparable to the relatively slow JCVI‐Syn3A replication rate of 100 bp/s were insufficient to produce chromosome partitioning within a cell cycle, even when up to 50 SMC complexes per chromosome and dwell times of 200 s were used.

One possible interpretation is that in our model, successful partitioning requires large enough SMC processivities (vτ), and the lower translocation speeds are unable to attain these loop sizes with the ranges of τ we tested. However, it is possible that the effective extrusion velocity used in our model represents the cumulative effect of many stochastic 3D DNA capture events rather than a simple 1D translocation process. Consistent with this picture, recent mechanistic models propose that SMC complexes sample DNA within an anisotropic search sector and occasionally capture DNA segments that are distant along the contour but proximal in three‐dimensional space (“trans‐grabbing”) (Bonato et al., 2025). Such stochastic capture events could be consistent with slower ~100 bp/s 1D motion of the SMC complex along DNA, while the occasional trans‐grabbing could produce long‐range contacts otherwise only achievable by faster translocation speeds in the purely 1D model.

A related observation arises from the comparison between simulated and experimental chromosome contact maps. Our simulated contact maps did not resemble the experimental 3C contact map unless SMC parameters were chosen such that the average loop length was comparable to the size of the entire genome (vτ/L∼1). For example, a translocation speed of 500 bp/s and a dwell time of τ=2500s (corresponding to an average loop length of ~1 Mb) produced relatively uniform contact maps. Similarly long dwell times (τ∼2500 s) have previously been reported to best recapitulate Hi‐C maps of *B. subtilis* (Brandão et al., 2021). However, such dwell times are one to two orders of magnitude larger than the ~1 min residence times observed in in vivo experiments (Kleine Borgmann et al., 2013; Mäkelä & Sherratt, 2020). Occasional inter‐strand capture events of the type proposed by Bonato et al. could generate long‐range contacts without requiring extremely long residence times, potentially explaining the relatively uniform contact maps observed experimentally.

Chromosome segregation necessarily requires the resolution of topological entanglements between sister chromosomes. In the present model, strand crossings are permitted through a reduction of excluded‐volume interactions between DNA segments, which serves as a simplified representation of type II topoisomerase activity. The JCVI‐Syn3A genome encodes homologs of both DNA gyrase (*gyrA*/*gyrB*) and topoisomerase IV (*parC*/*parE*), the primary enzymes responsible for relaxing supercoiling and resolving catenation in bacteria. Proteomics measurements indicate that these enzymes are present in substantial abundance in Syn3A, with estimated copy numbers of ~244 to 298 for gyrase subunits and ~49 to 157 for topoisomerase IV subunits across available datasets (Breuer et al., 2019; Brier et al., 2025). Contact maps of Syn3A chromosomes in stationary phase show no persistent signatures of supercoiling (Gilbert et al., 2021), suggesting that topoisomerase activity efficiently relaxes torsional stress and resolves DNA entanglements in vivo. In our simulations strand crossings occur during the minimization step following SMC updates, primarily as a computational approximation rather than as a mechanistic model of SMC–topoisomerase cooperation.

Recent polymer simulations of the *B. subtilis* chromosome have suggested that SMC activity alone can drive chromosome compaction and segregation. In these models, segregation still occurs in the absence of the ParABS system, although the process becomes less efficient (Brahmachari et al., 2024). This is broadly consistent with experimental observations showing that deletion of ParA or ParB in *B. subtilis* does not abolish chromosome partitioning, although it leads to defects such as delayed origin segregation and increased replication initiation (Lee & Grossman, 2006). Our results for JCVI‐Syn3A are qualitatively similar: efficient segregation can be achieved without a ParABS system, but only within an appropriate region of parameter space.

One important difference between these systems is chromosome size. The JCVI‐Syn3A genome (~543 kb) is nearly an order of magnitude smaller than the ~4.2 Mb genome of *B. subtilis*. Notably, the origin‐proximal region of the *B. subtilis* chromosome that contains eight of its 10 parS sites spans ~800 kb (Breier & Grossman, 2007), already larger than the entire JCVI‐Syn3A chromosome. As another point of reference, the size of the minimal cell chromosome is comparable to that of topologically associating domains (TADs) observed in larger genomes (Marin‐Gonzalez et al., 2025). These differences suggest that the physical scale of the chromosome may strongly influence the mechanisms required for segregation.

Another key difference may lie in the effective loop coverage achieved by SMC activity in different organisms. For a small genome such as JCVI‐Syn3A, loop extrusion by a modest number of SMC complexes can generate loops spanning a substantial fraction of the chromosome, thereby promoting efficient separation of replicated strands. In contrast, for larger genomes the same SMC parameters would yield substantially lower loop coverage. For example, estimates for *B. subtilis* suggest on the order of several tens of active SMC complexes per chromosome (Schibany et al., 2018), comparable to that which we estimate for JCVI‐Syn3A. Assuming *B. subtilis* and JCVI‐Syn3A share similar values of ν ~ 0.1–1 kbp/s and τ∼60s, the resulting loop coverage would be correspondingly smaller for *B. subtilis*. In such cases, additional partitioning systems such as ParABS may play a larger role in ensuring robust chromosome segregation. This interpretation is supported by recent polymer simulations (Harju et al., 2024).

## 4 CONCLUSIONS

We investigated the parameters of our chromosome polymer model to determine whether chromosome segregation could be achieved through SMC loop extrusion and topoisomerases without invoking a fictitious force, using the replication and cell‐division timings predicted by our previous 4DWCM. We performed a scan of SMC number (*N*), translocation speed (*v*), and dwell time (*τ*) and found that chromosome segregation in JCVI‐Syn3A can arise naturally from SMC‐driven loop extrusion when the loop coverage, defined as Nvτ/L, approaches order unity. This quantity, which can be interpreted as the approximate fraction of the chromosome extruded into loops, provides a useful organizing principle for understanding segregation outcomes. However, parameter sets with identical values of Nvτ/L but extreme combinations of N, v, and τ produced substantially different dynamics and often failed to complete segregation within a cell cycle. These results suggest that efficient partitioning occurs within a favorable region of parameter space rather than according to a single control parameter. Notably, parameter regimes close to experimentally inferred SMC properties consistently produced successful segregation in our simulations, whereas extreme regimes (e.g., *N*, *ν*, or *τ* much lower than experimentally observed/inferred values) that are unlikely to occur in vivo performed poorly.

Our simulations suggest that only a modest number of cellular SMC complexes need to be chromosome‐bound to achieve segregation, provided sufficient high translocation speeds and dwell times. If fluorescent tagging of SMC becomes feasible without disrupting function, our model predicts experimentally testable changes in SMC spatial organization, such as the appearance of three distinct SMC “traffic jams” when the chromosome is partially replicated, provided a sufficient number of bound SMC complexes and provided the SMC complexes block each other sufficiently long times. Finally, we find that short stall times between SMC complexes, effectively minimizing SMC–SMC blocking, and long SMC dwell times, produce the most uniform SMC distributions and yield contact maps in closest agreement with experiment. Dwell times within the range ~1 min observed in in vivo experiments do not result in uniform contact maps, but inter‐strand grabbing between DNA segments that are distant along the contour but proximal in three‐dimensional space may help explain the uniformity of experimentally observed contact maps without requiring extremely long dwell times. Additional factors, including DNA bead size, exclusion of ribosomes from the nucleoid, and electrostatic interactions, remain important directions for future investigation.

## 5 METHODS

### 5.1 Simulation of cell growth, chromosome replication, and chromosome segregation

The DNA replication state and cell geometry are based on the average behavior predicted by the 4DWCM, using the mean trace from 50 simulations (Figure 1). We assume that replication initiation occurs at 5 min into the cell cycle, replication completes at 50 min, the volume has doubled at 65 min, and the cell cycle completes at 105 min.

Chromosome replication is modeled using the replication dynamics predicted by the 4DWCM, in which the replication rate depends on the instantaneous deoxyribonucleotide triphosphate (dNTP) pools (Thornburg et al., 2026). In the full model, replication initiates when DnaA binds near the origin; for simplicity, we assume that initiation occurs 5 min into the cell cycle. Replication is modeled using the train track model (Gogou et al., 2021). Replication forks independently traverse the opposite arms of the mother chromosome, with beads for the left and right daughter chromosomes appearing in locations centered about the location of the mother's corresponding bead. We use a replication rate of 100 bp/s, updating each replication fork by 20 beads every 2 s. The replication rate used in our simulations (100 bp/s) is motivated by experimental measurements in *Mycoplasma*, originally reported for *Mycoplasma capricolum* (Seto & Miyata, 1998).

Cell growth and division follow the changes in surface area predicted by the 4DWCM (Figure 1), where surface area is computed from the number of lipids and membrane proteins. In the 4DWCM, cell surface area growth is determined by the accumulation of membrane lipids and membrane proteins, while the cell shape evolves during the cell cycle from spherical to prolate to dumbbell‐shaped before dividing into two spherical daughter cells. These morphological transitions are consistent with fluorescence imaging observations of dividing JCVI‐Syn3A cells (Thornburg et al., 2026).

We assume the cell grows as a sphere from 0 to 65 min, until it reaches twice its initial volume (corresponding to a radius of 252 nm). Because spherical growth implies that surface area increases more slowly than volume, this geometry predicts that the cell volume would reach twice its initial value before the surface area has doubled. However, by the end of the cell cycle both the volume and surface area must have doubled. To reconcile these constraints, we assume that the cell volume remains constant during this interval.

From 65 to 105 min, the geometry is modeled as two overlapping symmetric spheres whose radii and separation reproduce the surface areas and volumes in Figure 1. The vector between overlapping symmetric spheres is chosen to point in the same direction as the vectors between the centers of mass between daughter chromosomes. In the present work we do not explicitly model the mechanical coupling between chromosome segregation and membrane shape changes during division.

Chromosome dynamics simulations begin at 5 min, immediately after replication initiation. We chose to skip the first 5 min of the cell cycle to save computation time. The initial chromosome configuration is generated using sc_chain_generation (Gilbert et al., 2023) with a spherical cell radius of 206 nm.

Topoisomerases are implemented implicitly by momentarily removing excluded volume term (in practice we retain 0.2 $k_{B}T$ barrier height) during minimization of SMC loop bonds, which relaxes the chromosomes via decatenation of entanglements. SMC and replication are updated and topoisomerases are applied every 2 s of biological time, and a relaxation procedure described below in Section 2.2 is used. Every 1 min of biological time, a long equilibration is performed, the cell morphology is updated, and five new ribosomes are introduced at random locations within the cell.

The number of ribosomes and SMC are assumed to double over the course of the simulation. The number of ribosomes increases from 500 at the start of the simulation by adding 5 ribosomes per minute at random positions until the end of the cell cycle.

### 5.2 SMC loop extrusion model

We implemented a stochastic 1D loop extrusion model in which SMC complexes translocate along a replicating chromosome with lattice sites corresponding to 10 bp. Based on observations in vitro and computational predictions, we take the SMC extrusion to be bidirectional (Banigan et al., 2020; Banigan & Mirny, 2019; Barth et al., 2025) where each side of the loop translocates simultaneously with an extrusion speed of 50–250 bp/s for an effective translocation rate of 100–500 bp/s. Each SMC is represented by two motor heads that bind symmetrically and occupy a footprint of $\left(1+2w\right)$ bp, where w is the half‐width of the SMC footprint (here w=smcWidth=1). We keep the binding–unbinding dynamics restricted to a fixed pool of N SMCs, but progressively load an additional N SMCs during replication, so that the total number of chromosome‐bound SMCs increases from N to 2N over the course of DNA replication. The number of SMCs remains fixed after the completion of DNA replication. The number of bound SMCs (20–50) is based on proteomics measurements of Smc in JCVI‐Syn3A (Breuer et al., 2019; Brier et al., 2025), assuming that roughly half of the total Smc population is bound at any given time and that SMC complexes are formed as a dimer of Smc proteins. Proteomics counts of ScpA and ScpB were not used to determine the number of SMC complexes included in our model. The parameters used in our simulations are summarized in Table 1.

**TABLE 1:** Simulation parameters for structural maintenance of chromosomes (SMC) loop extrusion.

Parameter	Value used
Number of loops	Number of translocating SMC complexes per chromosome. We simulate *N* = 20–50. During the cell cycle the number of complexes increases from *N* at the start of the cycle to 2*N* by the end of DNA replication.
Extrusion speed (bp/s)	SMC complexes are modeled with an extrusion speed of 200–500 bp/s and rapid direction switching. Each side of the complex translocates 500 bp every 2 s, corresponding to 100–250 bp/s per arm.
SMC unloading rate (s^−1^)	Rate at which SMC complexes dissociate from the chromosome. In the parameter scan we simulate dwell times of 50–100 s (0.01–0.02 s^−1^). For stationary‐phase simulations we also consider a long dwell time of 2500 s (0.0004 s^−1^), with an increased unloading rate of one event per 200 s when SMC complexes become blocked, as well as a short dwell time of 4 s (0.25 s^−1^). For simulations testing enhanced unloading at replisomes we consider rates of one event per 25 s (0.04 s^−1^) and immediate unbinding.
SMC–replisome bypass rate (s^−1^)	SMC complexes are not allowed to bypass replication forks in the current model and are removed if overtaken by an advancing replication fork.
SMC–SMC bypass rate (s^−1^)	Rate at which one SMC complex can bypass another after a collision. We tested both immediate bypassing (no blocking) and blocking with a bypass rate of one event per 20 s.

#### 5.2.1 Loading and occupancy

In the absence of ParA, ParB, and canonical parS sites in JCVI‐Syn3A, and because the 3C contact map does not display a secondary diagonal indicative of origin‐proximal SMC loading, we assume that SMC complexes load randomly and uniformly along the chromosome in our simulations. SMCs loading is subject to two constraints: (i) the local neighborhoods of both heads must be unoccupied, and (ii) the positions must not overlap replication forks. SMC heads are initialized as being three lattice sites apart. Occupied sites are tracked with a binary array over the chromosome, where 1 marks an SMC‐bound site and 0 an unoccupied site. Before beginning the recorded simulation, we load SMCs onto the unreplicated chromosome and equilibrate the initial SMC positions in 1D for 360,000 steps. We do not equilibrate the chromosome conformation with SMC looping in 3D before the start of the simulation, although simulations with a 15 min topological pre‐stress showed no difference in partitioning behavior.

#### 5.2.2 Blocking and bypassing

Each step of the 1D loop extrusion algorithm corresponds to 40 ms of biological time. For each step of the algorithm, each SMC attempts to move its two heads in opposite directions (left and right) with probability step_prob=1.0. An attempt succeeds only if the target region is unoccupied and does not overlap a replication fork. If movement is blocked, the SMC is marked as stalled, and attempts to bypass the obstruction with probability bypass=0.002 (1 per 20 s), searching forward up to 15 bp for the nearest vacant location. If bypass does not occur, the motor remains stalled.

#### 5.2.3 Dissociation and reloading

SMCs dissociate stochastically. For parameter scan simulations, motors dissociate with rate $\text{basal}\_\text{death}\_\text{prob}=20\;\mathrm{bp}/\text{step}\;{\left(\mathrm{v\tau}\right)}^{-1}$. For stationary phase simulations with SMC–SMC blocking (Figure 8b), unstalled motors translocate at *ν* = 500 bp/s and dissociate with a basal rate basal_death_prob=0.000016 per step corresponding to 1 per 2500 s or a mean lifetime of ~2500 s, and stalled motors dissociate at a higher rate stall_death_prob=0.0002 or 1 per 200 s. For simulations without SMC–SMC blocking (Figure 8c), all SMC dissociate with a rate basal_death_prob=0.000016 per step, corresponding to 1 per 2500 s or a mean lifetime of ~2500 s. For simulations with short dwell time (Figure 8d), all SMC dissociate with a rate basal_death_prob=0.01 per step, corresponding to 1 per 4 s or a mean lifetime of ~4 s. For simulations with enhanced unloading when reaching the replisome on the daughter side, motors dissociate with rate $\text{basal}\_\text{death}\_\text{prob}=20/v\tau_{\text{repl}.}$ where $\tau_{\text{repl}.}=25\;s$. Upon dissociation, the SMC is immediately reloaded at a new valid position, ensuring the total SMC count remains constant. In the model, replication actively unloads any SMC whose position is overtaken by the advancing replication fork.

#### 5.2.4 Communication with polymer model

**FIGURE 10:**
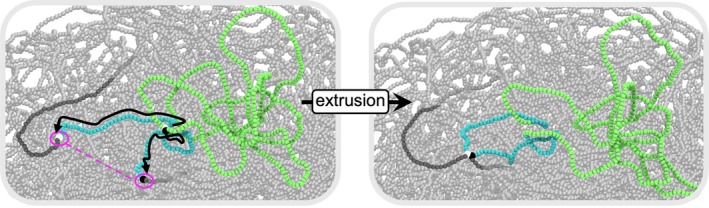
Renders illustrating loop formation before and after structural maintenance of chromosomes (SMC) translocation. In this example, the SMC is unblocked on both sides, permitting each side (white and black beads) to translocate the full distance of 50 beads (equivalent to 250 bp/s per side over 2 s). Newly extruded DNA is shown in cyan, the pre‐existing loop in lime, and the remainder of the chromosome in gray. When the positions of the sides of the SMC are updated, the harmonic bond between old SMC positions is removed and a new harmonic bond is introduced between the sides of the SMC at their new positions, shown as a magenta dashed line.

At every 2 s of biological time, replication of the chromosome is communicated from the btree_driver object in btree_chromo to the loop_simulator. New loops are added based on the extent of replication, and loops that a replication fork has passed are re‐initialized. Every 2 biological seconds, the simulator iterates by 50 steps (40 ms biological time per algorithm step). The choice of 50 steps corresponds to translocation of 250 bp on each side every 1 s, for an effective translocation rate of 500 bp/s if not stalled. The replication state and positions of all SMC are communicated to the LAMMPS object and the energy minimizations and equilibrations are performed. Renders for an example SMC translocating by 50 beads on each side is shown in Figure 10.

### 5.3 Polymer model and Brownian dynamics

JCVI‐Syn3A's chromosome is modeled as an elastic worm‐like chain with 10 bp per bead (Gilbert et al., 2023; Thornburg et al., 2026). Bonds between beads of the DNA backbone are harmonic using bond_style harmonic in LAMMPS:

$$U_{i}^{s}=\kappa_{s}{\left(r-r_{0}\right)}^{2}$$

with equilibrium distance $r_{0}$ is set to be $\sigma_{\mathrm{DNA}}=3.4\;\mathrm{nm}$ and spring constant $\kappa_{s}=1000k_{b}T/\sigma_{\mathrm{DNA}}^{2}$. Bonds between the two sides of an SMC (dashed magenta line in Figure 10) are harmonic with $r_{0}$ is set to be $\sigma_{\mathrm{DNA}}$ and $\kappa_{s}=k_{B}T$. The bending potential is implemented using angle_style cosine:

$$U_{i}^{b}=\kappa_{b}\left[1-\cos\left(\pi -\theta_{i}\right)\right],$$

where $\theta_{i}$ is the angle between DNA beads i and i+1 and $\kappa_{b}=l_{p}k_{B}T/\sigma_{\mathrm{DNA}}$ with persistence length $l_{p}=45\;\mathrm{nm}$.

Excluded volume effects between DNA strands are implemented via a soft potential using pair_style soft:

$$U_{\mathrm{ij}}^{e.v.}=A\left[1+\cos\left(\frac{\pi r_{\mathrm{ij}}}{r_{c}}\right)\right]\Theta \left(r_{c}-r_{\mathrm{ij}}\right),$$

where $r_{c}$ is the cutoff distance, $r_{\mathrm{ij}}$ is the distance between beads i and j, and A is the potential strength. For the energy minimizations (every 2 biological seconds), $r_{c}=\sigma_{\mathrm{DNA}}$ and strand crossings are permitted by setting $A=0.2k_{B}T$. For the equilibrations with 10.2 nm DNA diameter (every 2 biological seconds), $r_{c}=3\sigma_{\mathrm{DNA}}$ and $A=5k_{B}T$. For equilibrations with 3.4 nm DNA diameter (every 1 biological minute), $r_{c}=\sigma_{\mathrm{DNA}}$ and $A=5k_{B}T$.

Ribosomes are modeled as spheres with diameter $r_{\text{ribo}}=20\;\mathrm{nm}$ and interact with DNA via a soft potential with $r_{c}=r_{\text{ribo}}+r_{\mathrm{DNA}}$ and $A=k_{B}T$. Ribosome positions were fixed during energy minimizations.

Equilibrations are performed using BD using a custom version of fix brownian in LAMMPS compatible with the GPU. The equation of motion for each bead is given by $\gamma \left(\mathrm{dr}/\mathrm{dt}\right)=-\nabla U+R\left(t\right)$. The damping constant for DNA is $\gamma =3\pi \eta \sigma_{\mathrm{DNA},\text{ribo}}$ with dynamic viscosity of 1.2 Pa s (Thornburg et al., 2022). The noise term $R\left(t\right)$ obeys the fluctuation‐dissipation theorem with an assumed temperature of 310 K. We use a 25 ns timestep for integration. For each equilibration at a 10.2 nm DNA diameter (performed every 2 biological seconds), we ran 20,000 BD steps during spherical growth (5–65 biological minutes). From 65 to 105 biological minutes, we increased the number of BD steps by 4500/min to accommodate the rapid expansion of the dividing cell. For each equilibration at a 3.4 nm DNA diameter (performed every 1 biological minute), we performed 500,000 BD steps.

Boundary particles were implemented as beads with radius $r_{\text{bdry}}=10\;\mathrm{nm}$ that create a closed spherical/overlapping sphere shapes described in Section 3.1. Excluded volume between boundary particles and DNA use $r_{c}=r_{\text{bdry}}+r_{\mathrm{DNA}}$ and $A=k_{B}T$, and between boundary particles and ribosomes use $r_{c}=r_{\text{bdry}}+r_{\text{ribo}}$ and $A=k_{B}T$.

### 5.4 Contact map calculation

The 3C‐seq library was mapped as described in Gilbert et al. (2021). The 3C‐seq contact maps were normalized to be a doubly‐stochastic matrix using a matrix balancing procedure of Knight and Ruiz (2012), which ensures that the total contact probability over any given row and any given column sums to 1.

To produce the in silico contact maps, we split the genome into segments of 1 kb, which is roughly the size of fragments from restriction digestion and also the resolution we use to generate the in silico contact maps. We used a hard distance cutoff of 6 nm between beads (i.e., step function crosslinking function) which registers a count between segments $s_{i}$ and $s_{j}$ if any of the beads coordinates from $s_{i}$ are within 6 nm from any of the bead coordinates from $s_{j}$. A voxelized approach was used to detect contacts, since direct comparison is slow (O(N^2^), with *N* ~100 k). Multiple contacts between beads from the same two segments $s_{i}$ and $s_{j}$ were counted as a single count; thus, each replicate results in a binary contact matrix with elements of either zero or 1. For each replicate, every element on the diagonal is 1, since contacts always exist between bonded beads.

For the contact maps of stationary phase cells, the contact maps from 10 replicates across 10 time points were summed and then normalized using the matrix balancing procedure of Knight and Ruiz (2012). For the contact maps of growing cells, due to the low number of replicates (10) and total counts, we decided not to use a normalization procedure and instead generate plots based on the absolute counts per bin.

## AUTHOR CONTRIBUTIONS

**Andrew K. Maytin:** Writing – original draft; writing – review and editing; conceptualization; visualization; software; formal analysis; methodology; validation. **Benjamin R. Gilbert:** Conceptualization; software. **Zaida Luthey‐Schulten**: Writing ‐ original draft; writing ‐ review and editing; conceptualization; funding acquisition; supervision.

## Data Availability

An archive containing simulation scripts, analysis code, and representative output data is available on Zenodo at https://doi.org/10.5281/zenodo.19959420. The archive also provides container configuration files and dependencies required to reproduce the computational environment used in this study.
